# Oral Bisphosphonates and Risk of Atrial Fibrillation and Flutter in Women: A Self-Controlled Case-Series Safety Analysis

**DOI:** 10.1371/journal.pone.0004720

**Published:** 2009-03-06

**Authors:** Anthony Grosso, Ian Douglas, Aroon Hingorani, Raymond MacAllister, Liam Smeeth

**Affiliations:** 1 Department of Pharmacy, University College London Hospitals NHS Foundation Trust, London, United Kingdom; 2 Centre for Clinical Pharmacology, Division of Medicine, University College London, London, United Kingdom; 3 Department of Epidemiology & Population Health, London School of Hygiene & Tropical Medicine, London, United Kingdom; 4 Division of Population Health, University College London, London, United Kingdom; Lerner Research Institute, Cleveland Clinic, United States of America

## Abstract

**Background:**

A recent trial unexpectedly reported that atrial fibrillation, when defined as serious, occurred more often in participants randomized to an annual infusion of the relatively new parenteral bisphosphonate, zoledronic acid, than among those given placebo, but had limited power. Two subsequent population-based case-control studies of patients receiving a more established oral bisphosphonate, alendronic acid, reported conflicting results, possibly due to uncontrolled confounding factors.

**Methodology/Principal Findings:**

We used the United Kingdom General Practice Research Database to assess the risk of atrial fibrillation and flutter in women exposed to the oral bisphosphonates, alendronic acid and risedronate sodium. The self-controlled case-series method was used to minimise the potential for confounding. The age-adjusted incidence rate ratio for atrial fibrillation or flutter in individuals during their exposure to these oral bisphosphonates (n = 2195) was 1.07 (95% CI 0.94–1.21). The age-adjusted incidence rate ratio for alendronic acid (n = 1489) and risedronate sodium (n = 649) exposed individuals were 1.09 (95% CI 0.93–1.26) and 0.99 (95% CI 0.78–1.26) respectively. In *post-hoc* analyses, an increased risk of incident atrial fibrillation or flutter was detected for patients during their first few months of alendronic acid therapy.

**Conclusions/Significance:**

We found no robust evidence of an overall long-term increased risk of atrial fibrillation or flutter associated with continued exposure to the oral bisphosphonates, alendronic acid and risedronate sodium. A possible signal for an increase in risk during the first few months of therapy with alendronic acid needs to be re-assessed in additional studies.

## Introduction

Oral bisphosphonates are effective in the prevention of osteoporotic fractures.[1] However, a recent large international randomized trial (HORIZON study) unexpectedly reported that serious (defined as fatal, life-threatening or resulting in hospitalization or disability) atrial fibrillation (AF) occurred more frequently in participants randomized to an annual infusion of zoledronic acid than among those given placebo (1.3% vs. 0.5%; p<0.001) raising concerns that AF may be an unexpected adverse effect of zoledronic acid treatment specifically, or of bisphosphonate therapy in general.[2] Re-analysis of a previous trial also reported a ‘trend’ towards an increased risk of serious AF events among patients treated with oral alendronic acid compared with placebo (1.5% vs. 1.0%; p = 0.07) [3] and a recent population-based case-control study found that ‘ever-use’ of oral alendronic acid was associated with an increased risk of incident AF (odds ratio 1.86; 95% confidence interval [CI], 1.09–3.15).[4] However, these data conflict with results from other studies. For example, earlier placebo-controlled trials, one of zoledronic acid and one of risedronate sodium, found no excess risk of AF.[5] Moreover, another recently published population-based case-control study found no evidence that use of oral bisphosphonates increases the risk of AF and atrial flutter.[6]

Although evidence from randomized trials is less likely to be biased, the trials were not designed, or powered, to detect differences in the risk of AF. Case-control studies have better power but are prone to the effects of confounding. For example, osteoporosis affects older people who are at higher risk of AF and risk factors for AF, such as hyperthyroidism, also increase the risk of osteoporosis.[1] Therefore people prescribed and not prescribed bisphosphonates may well differ in terms of risk of AF, and these differences are likely to cause bias and confounding in observational studies. Thus considerable uncertainty remains and these concerns have led to a recent Europe-wide review of bisphosphonates and AF, including review of clinical trial data, spontaneous reports of suspected adverse drug reactions and published literature. As a result, it was recently announced that product information for the intravenous bisphosphonates, zoledronic acid and pamidronic acid, will be updated to include AF as a possible side-effect.[7] The risk of AF with alendronic acid is to remain under close scrutiny.[7]

We studied the association between oral bisphosphonates and AF and atrial flutter in women using the self-controlled case-series method [8], [9] on routinely collected information from the large United Kingdom (UK) General Practice Research Database (GPRD). This approach provides information on an appropriately large scale, using routine clinical data while minimizing the biases that may affect case-control studies.

## Methods

### Participants

The GPRD is the world's largest computerized database of anonymized longitudinal medical records from primary care. Currently data are being collected on over 3.6 million patients from around 450 primary care practices throughout the UK.[10] Female patients exposed to oral alendronic acid (10mg daily or 70mg weekly) or risedronate sodium (5mg daily or 35mg weekly) between 1^st^ December 2004 and 31^st^ December 2006 were included in the study. This comprised almost 400,000 person-years of observations from 187 general practices.

Eligible participants were those who had a first-ever recorded diagnosis of AF or atrial flutter within a pre-defined study window. Medical diagnoses in the GPRD are recorded using OXMIS (Oxford Medical Information Systems) and Read codes. OXMIS codes are based on the International Classification of Diseases (ICD) and Office of Population and Census Statistics (OPCS) operation codes. Read codes became the standard for diagnostic classification in the GPRD during 1998 so both codes were utilized in this study. Both arrhythmias were coded separately (14 codes) with the exception of two codes where they were combined. We therefore studied AF and atrial flutter as one composite endpoint. Nevertheless, most cases were probably AF as a previous study of patients recorded in a Danish National Registry with an incident diagnosis of AF or atrial flutter, showed that only 5% had pure atrial flutter.[11] Study start dates were derived using the latter of the individual practice's up-to-standard date (GPRD-defined quality marker based on assessment of completeness, continuity and plausibility of data recording in key areas) or the patient's first registration date. Study end dates were derived using the minimum of the patient's transfer out date or the practice's last collection date.

If patients had recently (within 6 weeks) consulted their general practitioner before their diagnosed event with symptoms that could possibly indicate an arrhythmia, such as palpitations, their date of onset was altered to the date of first symptom. Similarly, if drugs such as digoxin were initiated within 6 weeks prior to the incident arrhythmic event, the date of event was altered accordingly.

Individuals were excluded if they had received cardiac glycosides, amiodarone, sotalol, verapamil, diltiazem or a cardioversion more than 6 weeks prior to their event because it suggested that the arrhythmia may not be a new event. Cases were also excluded if their medical records indicated that the arrhythmia was likely to have been retrospectively recorded. For example, if the patients AF or atrial flutter was recorded along with other diagnoses on the day of a ‘new-patient’ or ‘well-person’ screen. We also excluded people whose only diagnostic entry for their event appeared when the general practice received a post-mortem report because we were concerned that the date recorded would not accurately reflect the date of the arrhythmia.

Approval for our study was given by the Medicines and Healthcare Regulatory Agency (MHRA) Independent Scientific Advisory Committee (ISAC) for Database Research.

### Procedures

We used the self-controlled case-series method which relies on intra-person comparisons in a population of exposed individuals who have had the outcome of interest. Incidence rate ratios (IRR) of the outcome of interest are derived comparing defined intervals during exposure relative to all other observed time periods for each person.[8], [9], [12]–[15]

**Figure 1:**
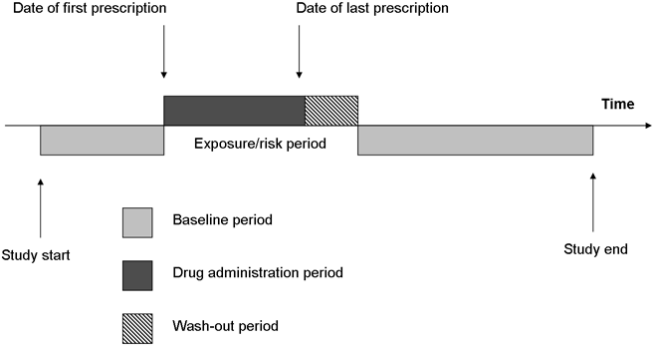
Pictorial representation of the self-controlled case-series method using total exposure time to define the ‘risk’ period.

The start of the exposed period was defined as the date of first bisphosphonate prescription. The end of the exposed period was defined as the date of the last prescription plus the final prescription quantity. A 30-day wash-out period was then added to the end of the exposure date to ensure significant drug elimination and to account for delays in obtaining prescriptions and pharmacy supplies. All other observation time within the study window was taken as the baseline (unexposed) period. Participants included had a least one prescription (exposure) for a bisphosphonate and at least one recorded episode of AF or atrial flutter (event). Figure 1 illustrates a single individual who had a single period of exposure to an oral bisphosphonate. The length of the exposed and baseline periods will usually vary for each participant.

### Statistical analysis

We controlled for age using ten, five-year, age bands (45–49 years, 50–54, 55–59 etc.). IRR and 95% CIs were calculated for incident events occurring within each stratum of the exposed period compared to baseline periods using the aforementioned case-series method. Sub-group analyses for alendronic acid and risedronate sodium exposed individuals were planned *a priori*. Data were analysed with Stata (*version 9.0; StataCorp LP, College Station, TX, USA*).

## Results

**Figure 2:**
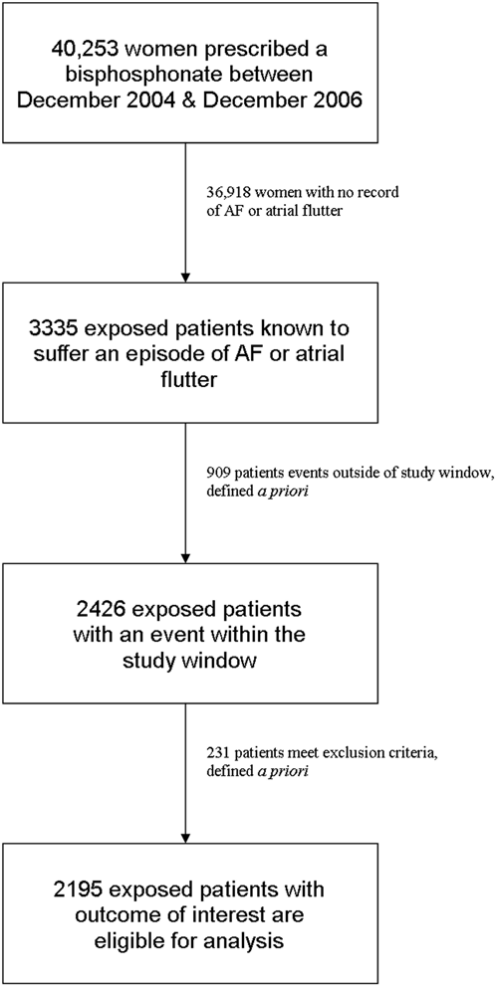
Flow-diagram indicating derivation of bisphosphonate study population.

40,253 female patients exposed to oral bisphosphonates were identified from the database, 3335 of whom were known to have had at least one recorded episode of either AF or atrial flutter. Figure 2 illustrates the derivation of the final study population that were eligible for analysis (n = 2195). The median age of women in the study was 82 years (interquartile range [IQR] 76–86) and the median total observation period (comprising in excess of 25,000 person-years) and exposure periods were 12.8 years (IQR 7.8–16.5) and 23.1 months (IQR 7.7–41.7) respectively. After controlling for age, the adjusted IRR for AF or atrial flutter was 1.07 (95% CI 0.94–1.21).

**Figure 3:**
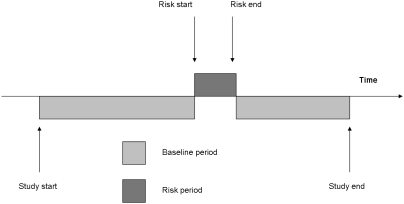
Time-to-event analysis using pre-defined ‘risk’ periods after commencement of therapy.

**Figure 4:**
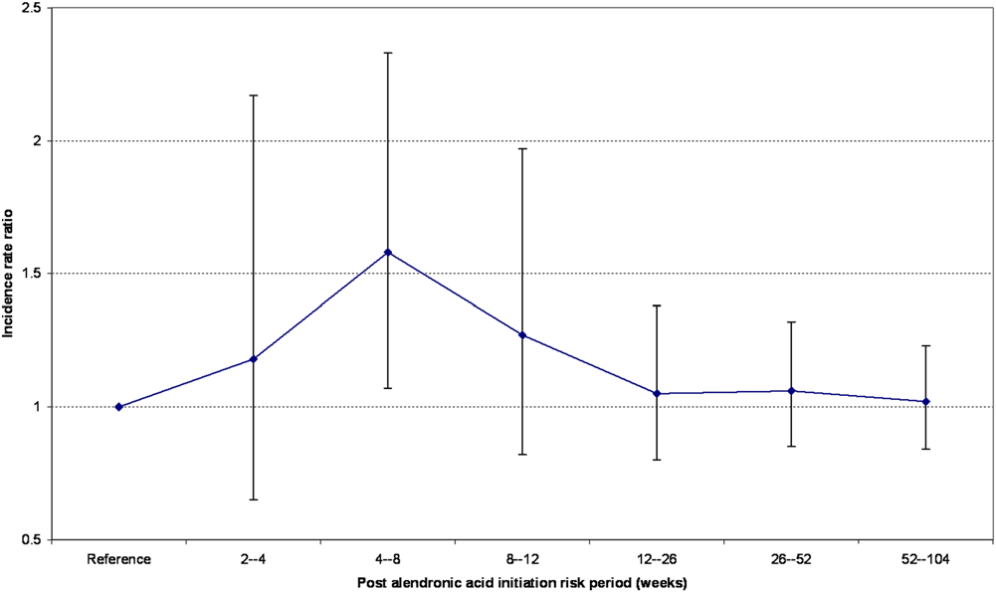
Risk estimates of incident atrial fibrillation or flutter after initiation of alendronic acid.

**Figure 5:**
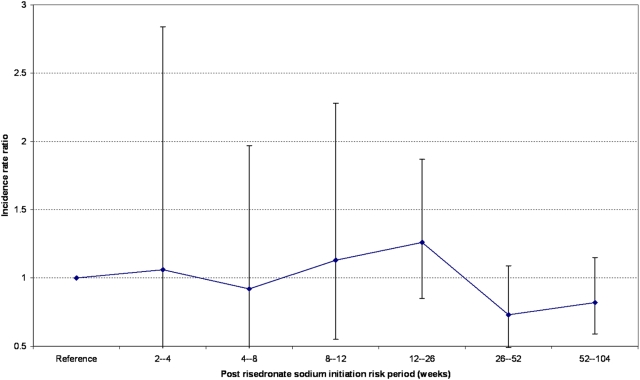
Risk estimates of incident atrial fibrillation or flutter after initiation of risedronate sodium.

We conducted sub-group analyses, specified *a priori*, for alendronic acid (n = 1489) and risedronate sodium (n = 649) exposed patients. Fifty-seven patients who had received both therapies were excluded. The calculated age-adjusted IRR for AF or atrial flutter was 1.09 (95% CI 0.93–1.26) and 0.99 (95% CI 0.78–1.26) for individuals exposed to alendronic acid and risedronate sodium respectively (see Table 1). In addition, we undertook *post-hoc* time-to-event analyses (see Figure 3) in order to determine if the risk of AF or atrial flutter differed by time after initiation of treatment (see Table 2). A signal for an increased risk of incident AF or atrial flutter was detected for patients during their first few months of alendronic acid therapy (see Figure 4) and no risk window was observed for risedronate sodium (see Figure 5).

**Table 1:** Risk of incident atrial fibrillation or flutter during exposure to the oral bisphosphonates, alendronic acid and risedronate sodium.

	N	n (baseline)	n (exposed)	IRR	95% CI
Total bisphosphonate population	2195	1457	738	1.07	0.94–1.21
Alendronic acid exposed individuals	1489	960	529	1.09	0.93–1.26
Risedronate sodium exposed individuals	649	474	175	0.99	0.78–1.26

N = number or participants; n = number of events; IRR = incidence rate ratio; CI = confidence interval

**Table 2:** Risk of incident atrial fibrillation or flutter after initiation of bisphosphonate therapy.

Post bisphosphonate initiation ‘risk’ period	n (exposed)	n (baseline)	IRR	95% CI
2–4 weeks	16	2179	1.20	0.73–1.98
4–8 weeks	35	2160	1.39	0.99–1.96
8–12 weeks	31	2164	1.20	0.83–1.74
12–26 weeks	89	2106	1.11	0.89–1.39
26–52 weeks	121	2074	0.97	0.80–1.17
52–104 weeks	177	2018	0.94	0.80–1.11
**Post alendronic acid initiation ‘risk’ period**	**n (exposed)**	**n (baseline)**	**IRR**	**95% CI**
2–4 weeks	11	1478	1.18	0.65–2.17
4–8 weeks	27	1462	1.58	1.07–2.33
8–12 weeks	22	1467	1.27	0.82–1.97
12–26 weeks	58	1431	1.05	0.80–1.38
26–52 weeks	91	1398	1.06	0.85–1.32
52–104 weeks	133	1356	1.02	0.84–1.23
**Post risedronate sodium initiation ‘risk’ period**	**n (exposed)**	**n (baseline)**	**IRR**	**95% CI**
2–4 weeks	4	645	1.06	0.39–2.84
4–8 weeks	7	642	0.92	0.43–1.97
8–12 weeks	8	641	1.13	0.55–2.28
12–26 weeks	29	620	1.26	0.85–1.87
26–52 weeks	26	623	0.73	0.49–1.09
52–104 weeks	41	608	0.82	0.59–1.15

n = number of events; IRR = incidence rate ratio; CI = confidence interval.

We also conducted sensitivity analyses by increasing the wash-out period to two and three months. In addition, we also excluded patients whose observation period ended within one month of their event in case observation censoring, due to events such as death, biased the results. These sensitivity analyses yielded no material change in the point estimate for alendronic acid or risedronate sodium.

As a test of the robustness of the null result in relation to the overall risk of AF or atrial flutter, we also examined the risk of upper gastrointestinal problems, such as oesophagitis, in individuals exposed to alendronic acid (n = 5017), within the first three months of therapy, as this is considered an established adverse effect of alendronic acid treatment. Exposure to alendronic acid was associated with an increase in the risk of upper gastrointestinal symptoms (IRR = 1.26; 95% CI 1.04–1.51) which is in-keeping with other real life observational data relating to alendronic acid and upper gastrointestinal problems.[16]

## Discussion

We report the largest observational study to date investigating the association of oral bisphosphonates and AF involving over two thousand exposed patients with one or more recorded episodes of AF or atrial flutter. We found no evidence of an overall long-term increased risk of AF or atrial flutter associated with alendronic acid or risedronate sodium. However we could not exclude a small increase in risk of arrhythmia during the first few months of alendronic acid therapy. This apparent signal of an increased risk should be interpreted with caution because we had reduced statistical power to detect increases in risk over short time-frames in this analysis. Moreover, as there are no established biological mechanisms that might link bisphosphonate therapy to cardiac arrhythmia, it is also difficult to know if this signal of an increased risk soon after initiation of therapy has biological plausibility. In the HORIZON trial, the risk of AF was distributed uniformly over time, with the vast majority of events occurring more than 30 days after infusion, by which time zoledronic acid is undetectable in the circulation.[2] Alterations in serum calcium levels could be related to AF, but the administration of zoledronic acid had little or no effect on serum calcium levels measured 9 to 11 days after infusion.[2]

One major limitation of research using routinely collected clinical data is the robustness of the recording information, although diagnostic codes for AF have been validated within the GPRD with over 95% of cases confirmed by a questionnaire.[17] However a recent study indicated that many primary care professionals cannot accurately detect AF on an electrocardiogram.[18] Though we used AF/atrial flutter as a combined end-point, other European Registry data suggest that 95% of these cases are likely to be AF.[11]

Certain other important limitations also need to be borne in mind. First, secondary care prescriptions are unavailable from the sampling frame and this may have introduced a small degree of error in ascertaining the start of some exposure periods. In addition, we assumed that all patients actually took their medication as prescribed which is unlikely, especially for prophylactic medicines. Second, as in any study based on clinical identification of AF, there may have been a delay between onset of the arrhythmia, clinical presentation, confirmation of diagnosis and recording in GPRD. This could possibly have produced a bias towards the null, but would be unlikely to have obscured entirely a clinically meaningful effect. Third, our study which utilized anonymized patient data was unable to distinguish more ‘serious’ episodes of AF as reported in the randomized trial data. Fourth, data pertaining to other oral bisphosphonates, such as disodium etidronate and ibandronic acid, were not available in our supplied data set therefore these results are not directly applicable to these agents.

However, there were also several strengths to the approach we used and were also able to detect an increase in the risk of upper gastrointestinal problems, which is an established adverse effect of alendronic acid therapy in routine clinical practice. Research using the GPRD has the great advantage of its large size which means that we were able to include many more cases of AF in bisphosphonate exposed individuals than previous studies. Our data are consistent with the larger of the two recently published conflicting population-based studies which included 435 and 47 exposed patients with AF respectively [4], [6] and is in-keeping with a recent re-analysis of a large placebo controlled trial involving risedronate sodium.[5] The self-controlled case-series method we used also helps minimize confounding and other biases inherent in the more widely-used research designs in pharmacovigilance. Case-control studies can be very successful in identifying adverse effects from a new treatment where the risk is large, and the adverse effect being studied is otherwise rare in the group being treated. The risk of phocomelia from thalidomide exposure is one such example. However, detecting a small increase in the risk of an adverse event that is common among the patient group receiving a new treatment can be more challenging. AF is common among the elderly population who are the major users of bisphosphonate treatment and a non-causal association between bisphosphonate treatment and AF could arise by confounding because several risk factors for osteoporosis and AF are shared; and osteoporosis may itself be a risk factor for cardiovascular disease.[1] In this situation, the size of the error introduced by confounding could be similar in magnitude to the risk of the adverse effect. Biases and confounding are minimized in randomized trials but these are usually designed and powered as trials of efficacy, which means that there are often only few recorded adverse events in any one trial. This can lead to considerable uncertainty around the risk estimates derived. In contrast, the self-controlled case-series method has the advantage that confounding is minimized by ensuring the comparisons are intra-person. In other words, such an analysis removes the variation between individuals in risk factors for cardiac disease and thus fixed confounders are implicitly controlled for. Statistical techniques can control for confounders in traditional observational studies however these need to be known and measurable. Including multiple variables also subjects the regression model to the degree of uncertainty associated with each. Using recorded blood pressure is one such example. The self-controlled case-series method also requires only a sample of the cases (e.g. individuals exposed to oral bisphosphonates with a recorded episode of AF or atrial flutter), and thus avoids the need for selecting adequate controls. A similar technique, known as the case-crossover,[19] can also control for fixed confounders, however this method requires the assumption that exposure distribution in successive time periods is exchangeable.[20] The self-controlled case-series method we utilized does not require such an assumption. In particular, age or time effects can be allowed for in much the same way as in a cohort study.[20] Finally, when applied to data sets such as the GPRD, the risk information obtained relates to routine clinical use of a drug and therefore has good external validity.

In conclusion, we found no evidence of an overall long-term increased risk of AF or atrial flutter associated with the oral bisphosphonates, alendronic acid and risedronate sodium. These observational data, obtained using the self-controlled case-series method, are larger in scale and less prone to confounding than previous observational studies and provide reassurance that the overall long-term risk of AF or atrial flutter with the chronic use of alendronic acid and risedronate sodium is either very small or not present at all. The signal we detected for a possible increased risk during the first few months of alendronic acid therapy warrants replication and further clarification to assess its robustness.

## References

[1] Majumdar SR (2008). Oral bisphosphonates and atrial fibrillation.. BMJ.

[2] Black DM, Delmas PD, Eastell R, Reid IR, Boonen S (2007). Once-yearly zoledronic acid for treatment of postmenopausal osteoporosis.. N Engl J Med.

[3] Cummings SR, Schwartz AV, Black DM (2007). Alendronate and atrial fibrillation.. N Engl J Med.

[4] Heckbert SR, Li G, Cummings SR, Smith NL, Psaty BM (2008). Use of alendronate and risk of incident atrial fibrillation in women.. Arch Intern Med.

[5] Karam R, Camm J, McClung M (2007). Yearly zoledronic acid in postmenopausal osteoporosis.. N Engl J Med.

[6] Sorensen HT, Christensen S, Mehnert F, Pedersen L, Chapurlat RD (2008). Use of bisphosphonates among women and risk of atrial fibrillation and flutter: population based case-control study.. BMJ.

[7] (2008). MHRA Drug Safety Update July 2008; Vol 1, issue 12: 4.. MHRA.

[8] Farrington P, Pugh S, Colville A, Flower A, Nash J (1995). A new method for active surveillance of adverse events from diphtheria/tetanus/pertussis and measles/mumps/rubella vaccines.. Lancet.

[9] Whitaker H (2008). The self controlled case series method.. BMJ.

[10] General Practice Research Database.. http://www.gprd.com/home/.

[11] Frost L, Vestergaard P (2004). Alcohol and risk of atrial fibrillation or flutter: a cohort study.. Arch Intern Med.

[12] Farrington CP, Nash J, Miller E (1996). Case series analysis of adverse reactions to vaccines: a comparative evaluation.. Am J Epidemiol.

[13] Farrington CP (1995). Relative incidence estimation from case series for vaccine safety evaluation.. Biometrics.

[14] Hubbard R, Lewis S, West J, Smith C, Godfrey C (2005). Bupropion and the risk of sudden death: a self-controlled case-series analysis using The Health Improvement Network.. Thorax.

[15] Grosso A, Douglas I, Hingorani A, MacAllister R, Smeeth L (2008). Post-marketing assessment of the safety of strontium ranelate; a novel case-only approach to the early detection of adverse drug reactions.. Br J Clin Pharmacol.

[16] Donahue JG, Chan KA, Andrade SE, Beck A, Boles M (2002). Gastric and duodenal safety of daily alendronate.. Arch Intern Med.

[17] Ruigomez A, Johansson S, Wallander M, Rodriguez L (2002). Incidence of chronic atrial fibrillation in general practice and its treatment pattern.. Journal of Clinical Epidemiology.

[18] Mant J, Fitzmaurice DA, Hobbs FDR, Jowett S, Murray ET (2007). Accuracy of diagnosing atrial fibrillation on electrocardiogram by primary care practitioners and interpretative diagnostic software: analysis of data from screening for atrial fibrillation in the elderly (SAFE) trial.. BMJ.

[19] Maclure M (1991). The case-crossover design: a method for studying transient effects on the risk of acute events.. Am J Epidemiol.

[20] Whitaker H, Farrington CP, Spiessens B, Musonda P (2006). Tutorial in biostatistics: the self-controlled case series method.. Stat Med.

